# LRRK2 Regulates Voltage-Gated Calcium Channel Function

**DOI:** 10.3389/fnmol.2016.00035

**Published:** 2016-05-23

**Authors:** Cade Bedford, Catherine Sears, Maria Perez-Carrion, Giovanni Piccoli, Steven B. Condliffe

**Affiliations:** ^1^Department of Physiology, University of OtagoDunedin, New Zealand; ^2^Center for Integrative Biology (CIBIO), University of TrentoTrento, Italy; ^3^Dulbecco Telethon InstituteTrento, Italy

**Keywords:** calcium channels, LRRK2, calcium signaling, protein interactions, Parkinson’s disease

## Abstract

Voltage-gated Ca^2+^ (Ca_V_) channels enable Ca^2+^ influx in response to membrane depolarization. Ca_V_2.1 channels are localized to the presynaptic membrane of many types of neurons where they are involved in triggering neurotransmitter release. Several signaling proteins have been identified as important Ca_V_2.1 regulators including protein kinases, G-proteins and Ca^2+^ binding proteins. Recently, we discovered that leucine rich repeat kinase 2 (LRRK2), a protein associated with inherited Parkinson’s disease, interacts with specific synaptic proteins and influences synaptic transmission. Since synaptic proteins functionally interact with Ca_V_2.1 channels and synaptic transmission is triggered by Ca^2+^ entry via Ca_V_2.1, we investigated whether LRRK2 could impact Ca_V_2.1 channel function. Ca_V_2.1 channel properties were measured using whole cell patch clamp electrophysiology in HEK293 cells transfected with Ca_V_2.1 subunits and various LRRK2 constructs. Our results demonstrate that both wild type (wt) LRRK2 and the G2019S LRRK2 mutant caused a significant increase in whole cell Ca^2+^ current density compared to cells expressing only the Ca_V_2.1 channel complex. In addition, LRRK2 expression caused a significant hyperpolarizing shift in voltage-dependent activation while having no significant effect on inactivation properties. These functional changes in Ca_V_2.1 activity are likely due to a direct action of LRRK2 as we detected a physical interaction between LRRK2 and the β3 Ca_V_ channel subunit via coimmunoprecipitation. Furthermore, effects on Ca_V_2.1 channel function are dependent on LRRK2 kinase activity as these could be reversed via treatment with a LRRK2 inhibitor. Interestingly, LRRK2 also augmented endogenous voltage-gated Ca^2+^ channel function in PC12 cells suggesting other Ca_V_ channels could also be regulated by LRRK2. Overall, our findings support a novel physiological role for LRRK2 in regulating Ca_V_2.1 function that could have implications for how mutations in LRRK2 contribute to Parkinson’s disease pathophysiology.

## Introduction

Voltage-gated calcium (Ca_V_) channels play critical roles in cell signaling by enabling Ca^2+^ influx in response to membrane depolarization. Ca_V_2.1 channels, composed of the pore-forming α1A subunit and accessory β and α2δ subunits, are predominantly expressed in neuronal (Westenbroek et al., 1998) and endocrine cells (Mahapatra et al., 2012), clustered in subcellular locations specialized for secretion such as the presynaptic membrane of neurons and the secretory pole of endocrine cells. Here, Ca_V_2.1 channels provide a rapid, localized increase in intracellular Ca^2+^ that stimulates vesicle fusion, driving the physiological processes of neurotransmission (Qian and Noebels, 2001) and hormone release (Braun et al., 2008).

To achieve the correct spatial and temporal dynamics required for these processes, Ca_V_2.1 channel activity is stringently controlled by a variety of signaling proteins that interact with intracellular domains of the channel. These include SNARE proteins of the secretion machinery themselves which inhibit Ca_V_2.1 activity to restrict Ca^2+^ influx to sites of full core-complex assembly (Bezprozvanny et al., 1995; Cohen-Kutner et al., 2010; Condliffe et al., 2010). Ca^2+^ binding proteins such as calmodulin (DeMaria et al., 2001), parvalbumin and calbindin (Kreiner and Lee, 2006) also participate in the negative feedback function of Ca^2+^-dependent inactivation, down-regulating Ca_V_2.1 activity when intracellular Ca^2+^ increases. In addition, several protein kinases have been shown to interact with Ca_V_2.1 channel subunits to control channel activity. These include protein kinase C (Zamponi et al., 1997), Ca^2+^ calmodulin-dependent kinase II (Jiang et al., 2008) and glycogen synthase kinase-3 (Zhu et al., 2010). Not surprisingly, genetic defects in Ca_V_2.1 channels result in a variety of neurological diseases including migraine, ataxia and epilepsy (Rajakulendran et al., 2012). While many of these disease associated mutations result in a direct loss- or gain-of-function of the channel, recent evidence indicates that some mutations also disrupt regulatory interactions with signaling proteins (Serra et al., 2010; Condliffe et al., 2013).

Leucine rich repeat kinase 2 (LRRK2) is a large, multi-domain protein belonging to the ROCO family of proteins. Originally identified in screens for genes mutated in familial Parkinson’s Disease (Paisán-Ruiz et al., 2004; Zimprich et al., 2004), the pathophysiological effects of mutations in LRRK2 have been the focus of many studies (Li et al., 2009; Dusonchet et al., 2011; Martin et al., 2014). In contrast, less is known regarding the normal physiological role of LRRK2. Potential roles have been described for autophagy (Alegre-Abarrategui et al., 2009; Tong et al., 2010), vesicle trafficking (Piccoli et al., 2011; MacLeod et al., 2013) and cytoskeleton dynamics (Meixner et al., 2011; Law et al., 2014) where LRRK2 has been shown to target important molecular components of these pathways. A growing body of evidence also supports a role for LRRK2 in Ca^2+^ signaling and homeostasis. LRRK2 was found to activate a Ca^2+^/CaMKK/AMPK pathway to increase formation of autophagosomes which could be blocked by buffering cytosolic Ca^2+^ (Gómez-Suaga et al., 2012). Cherra et al. (2013) demonstrated that neurons expressing mutant LRRK2 had reduced cytosolic Ca^2+^ buffering capacity and enhanced mitochondrial degradation that could be restored through calcium chelation or inhibition of Ca_V_ channels. Recently, LRRK2 was found to up-regulate Na^+^/Ca^2+^ exchanger expression and activity to influence Ca^2+^ dynamics in dendritic cells (Yan et al., 2015).

Based on our previous work showing a presynaptic role for LRRK2 via interactions with SNARE proteins and functional effects on neurotransmission (Piccoli et al., 2011; Cirnaru et al., 2014), we hypothesize that LRRK2 also targets the major presynaptic Ca^2+^ influx pathway, the Ca_V_2.1 channel, as a further mechanism to modulate intracellular Ca^2+^ homeostasis. This was investigated in the present study using patch-clamp electrophysiology to characterize how both heterologous and endogenous Ca_V_ channel activity is affected by expression of various LRRK2 constructs. We further analyzed a potential interaction between Ca_V_ channel subunits and LRRK2 via co-immunoprecipitation.

## Materials and Methods

### Cell Culture and Transfection

HEK293 cells were grown in a humidified atmosphere of 5% CO_2_ at 37°C in DMEM with 10% FBS, 1% penicillin/streptomycin and 1% glutamine. PC12 cells were maintained under the same conditions in RPMI-1640 medium supplemented with 10% horse serum, 5% FCS and 1% penicillin/streptomycin. For electrophysiology experiments, both cell types were plated on glass coverslips pre-treated with poly-L-lysine. cDNA encoding for the human Ca_V_2.1 α1A subunit (NM_001127222) was cloned in pEGFP-C1 to produce an α1A subunit with an EGFP tag at the c-terminus as described previously (Condliffe et al., 2013). In this prior study, we characterized the protein expression, trafficking and function of the tagged channel. The rat β3 subunit DNA (NM_001127222) was cloned in pCMV6 and rabbit α2δ1 (NM_001082276) was cloned in pcDNA3.1. At 24 h after plating, HEK293 cells were then transfected with the EGFP-tagged α-1A Ca_V_2.1 channel subunit together with the accessory Ca_V_ subunits β3 and α2δ1 in a 1:2:2 molar ratio using Lipofectamine 2000 (Invitrogen). HEK293 cells were co-transfected with wild-type (wt) LRRK2, G2019S LRRK2 or wt LRRK1 (1:2:2:1 molar ratio; Table 1) while PC12 cells were transfected with either EGFP alone or EGFP + wt LRRK2. Human LRRK2 (NM_198578.3) was cloned in pcDNA3.0 backbone as described in Gloeckner et al. (2006) while human LRRK1 (NM_024652.4) was cloned in pCMV-2×Myc vector as described in Greggio et al. (2007).

**Table 1:** **Biophysical properties of HEK293 cells recorded in different experimental conditions**.

Transfection/treatment conditions	*I*_max_ (pA) (mean ± SEM)	Cm (pF) (mean ± SEM)	Number of cells	Number of independent experiments
Ca_V_2.1 (α1A, β3, α2δ1)	−396.5 ± 90.1	30.9 ± 7.1	12	5
+LRRK2	−763.6 ± 97.9	31.9 ± 3.5	12	5
+LRRK1	−383.1 ± 85.7	39.3 ± 5.7	10	4
+G2019S	−992.0 ± 222.0	26.6 ± 3.8	9	4
+DMSO	−343.8 ± 64.3	38.5 ± 5.8	10	4
+LRRK2-IN-1	−401.5 ± 100.0	33.8 ± 6.1	10	4

### Solutions and Reagents

To inhibit LRRK2 kinase activity in transfected HEK293 cells, the LRRK2 kinase inhibitor LRRK2-IN-1 (Merck; Deng et al., 2011) was added to the cell culture medium 2 h before electrophysiological recordings at a final concentration of 1 μM. LRRK2-IN-1 was also added to the external bath solution at the same concentration for recordings from these pre-treated cells. The internal patch pipette solution for both HEK293 and PC12 electrophysiological recordings contained 120 mM ${\text{CsMeSO}}_{4}^{-}$, 10 mM CsCl, 10 mM HEPES, 4 mM Mg-ATP, 3 mM Tris-GTP, 2 mM MgCl_2_ and 1 mM EGTA (pH adjusted to 7.2 with CsOH). HEK293 cells were bathed in an external solution containing 145 mM tetraethylammonium chloride, 10 mM HEPES and 5 mM CaCl_2_. The external solution for PC12 recordings contained 115 mM NaCl, 4 mM KCl, 1 mM MgCl_2_, 10 BaCl_2_, 10 mM tetraethylammonium chloride, 10 mM glucose and 10 mM HEPES. TTX was added to this solution immediately prior to recordings at a final concentration of 1 μM to block endogenous voltage-gated Na^+^ currents.

### Whole Cell Patch Clamp Electrophysiology

Coverslips containing transfected HEK293 or PC12 cells were transferred to a perfusion chamber containing the appropriate external bathing solution mounted on an IX71 inverted fluorescence microscope (Olympus). Whole cell Ca_V_ currents were recorded from EGFP positive cells using patch pipettes prepared with a P-2000 puller (Sutter) to resistances measuring 2–3 megohms when filled with internal solution. Peak whole cell Ca^2+^ or Ba^2+^ currents were measured using an Axon Axopatch 200B amplifier (Molecular Devices) interfaced to a PC via a Digidata 1320 (Molecular Devices). Series resistance was routinely compensated at 60–80% and access resistance continually monitored during experiments where cells with uncompensated voltage errors >5 mV were excluded from analysis. Data were acquired at 10 kHz with leak and capacitative transients subtracted online with a P/4 protocol using pClamp 10.0 software. Current-voltage (*I-V*), activation and inactivation curves were acquired and fit with modified Boltzmann functions as described previously (Condliffe et al., 2013).

### Immunoblotting and Co-Immunoprecipitation

To evaluate exogenous LRRK1, wt LRRK2, G2019S LRRK2 and Ca_V_2.1 channel subunit protein expression in HEK293 cells as well as LRRK2 silencing in PC-12 cells, transfected cells were solubilized in lysis buffer (150 mM NaCl, 2 mM EDTA, 50 mM Tris-HCl, 1% NP-40 and 0.25% sodium deoxicolate, pH 7.4) by mechanical disaggregation and protein amount evaluated according to Bradford’s protocol. About 40 μg of total protein was diluted in 25% v/v Laemmli buffer 5×.

For endogenous LRRK2 immunoprecipitation, littermate wt and heterozygous BAC G2019S (Melrose et al., 2010) adult mouse forebrains were cross-linked by incubation with 0.8% formaldehyde solution for 2 h under agitation and then extensively washed with cold glycine 0.1 M. Brain tissue were then solubilized in lysis buffer (150 mM NaCl, 2 mM EDTA, 50 mM Tris-HCl, 1% NP-40 and 0.25% sodium deoxicolate, pH 7.4) by mechanical disaggregation and protein amount evaluated according to Bradford’s protocol. One milligram of total protein was incubated with 3 μg of rat anti-LRRK2 (clone 24D8, generous gift from Dr. Johannes Gloeckner) or rat anti-IgG (Abcam). Immunocomplexes were precipitated using protein G-Sepharose beads (GE-Healthcare, Freiburg, Germany) and eluted in Laemmli buffer at 55°C for 10 min. All samples were loaded onto a 10% SDS-PAGE and transferred into nitrocellulose membrane at 82V for 2 h at 4°C. The primary antibodies used were: rabbit anti-LRRK2 at 1:500 (MJFF2, c41-2 Abcam), rabbit anti-calcium channel β3 subunit 1:200 (Sigma), rabbit anti-calcium channel α1a subunit 1:200 (Sigma), mouse anti-actin 1:2000, mouse anti-myc 1:1000 (Sigma), rabbit anti-S6 ribosomal protein (S6RP) 1:1000 (Cell Signalling) applied in blocking buffer (20 mM Tris, pH 7.4, 150 mM NaCl, 0.1% Tween 20) and 5% nonfat dry milk, overnight at 4°C. The secondary antibody HRP-conjugated anti-rabbit (Jackson Immunoresearch) was used at 1:7000 dilution. Proteins were detected using the ECL detection system (GE Healthcare).

Housing and handling of mice were done in compliance with the guidelines established by the European Community Council (Directive 2010/63/EU of March 4th, 2014) and approved by the Italian Ministry of Health (IACUC 625).

### Data Analysis

All data are expressed as the mean ± SEM of *n* experiments. Statistical significance between two groups was determined using an unpaired *t*-test at the *p* level indicated. ANOVA was used for multiple comparisons with a *post hoc* Tukey’s test to identify significant differences between groups at the *p* level indicated.

## Results

### LRRK2 Specifically Increases Ca_V_2.1 Function

**Figure 1:**
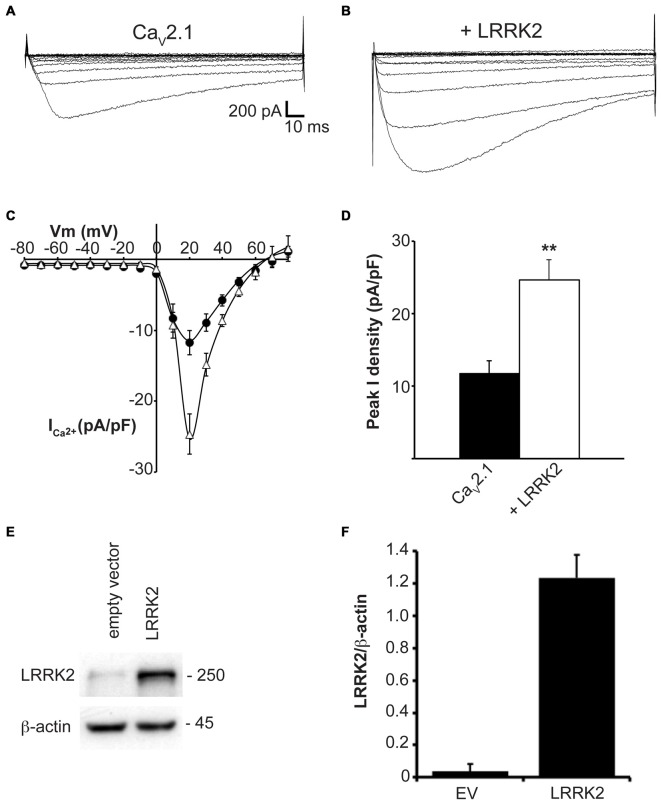
**LRRK2 increases Ca_V_2.1 current density.** Representative whole cell Ca^2+^ currents in response to a 10 mV voltage-step protocol recorded from HEK293 cells transfected with Ca_V_2.1 channels alone **(A)** or together with LRRK2 **(B)**. **(C)** Mean *I-V* relationships of peak Ca^2+^ current density from Ca_V_2.1 transfected cells in the absence (black circles) or presence (white triangles) of LRRK2 (*n* = 12 cells from five independent experiments). **(D)** Mean peak Ca^2+^ current density at a holding potential of 20 mV in cells expressing Ca_V_2.1 alone or co-expressing LRRK2 where ** indicates *p* < 0.01; unpaired *t*-test (*n* = 12 cells from five independent experiments). **(E)** HEK293 cells were transfected with empty vector (EV) or wild-type (wt) LRRK2 and expression of LRRK2 was evaluated by western-blotting. **(F)** The graph reports LRRK2 protein level normalized to the level of endogenous β-actin. Data are expressed as mean ± SEM, (*n* = 8).

To determine whether LRRK2 was able to modulate Ca_V_2.1 channel function, whole cell Ca^2+^ currents were recorded from HEK293 cells co-expressing LRRK2 and Ca_V_2.1 channel subunits. Representative *I-V* traces generated using a voltage-step protocol demonstrate that larger inward Ca^2+^ currents were recorded from LRRK2 expressing cells compared to cells transfected only with all three Ca_V_2.1 channel subunits (Figures 1A,B; Table 1). Comparison of the mean *I-V* relationships shows an increased Ca^2+^ current density in LRRK2 cells across a range of depolarized voltages (Figure 1C). While LRRK2 increased peak current density, it did not cause a detectable voltage shift in the *I-V* relationship. Peak Ca^2+^ current density in both LRRK2 and control cells was observed at 20 mV (Figure 1D), however transfection of LRRK2 resulted in a significantly increased Ca^2+^ current density at this voltage of −24.65 ± 2.81 pA/pF (*n* = 12) compared to −11.76 ± 1.76 pA/pF (*n* = 12) in control cells. To confirm LRRK2 overexpression, the amount of LRRK2 protein was measured via western blotting from HEK293 lysates transfected with empty vector (EV) or LRRK2 (Figure 1E). Normalized to endogenous β-actin expression, results show an elevated level of LRRK2 protein in LRRK2 transfected cells relative to EV controls (Figure 1F). Altogether, these results demonstrate that overexpression of LRRK2 augments Ca_V_2.1 channel function.

**Figure 2:**
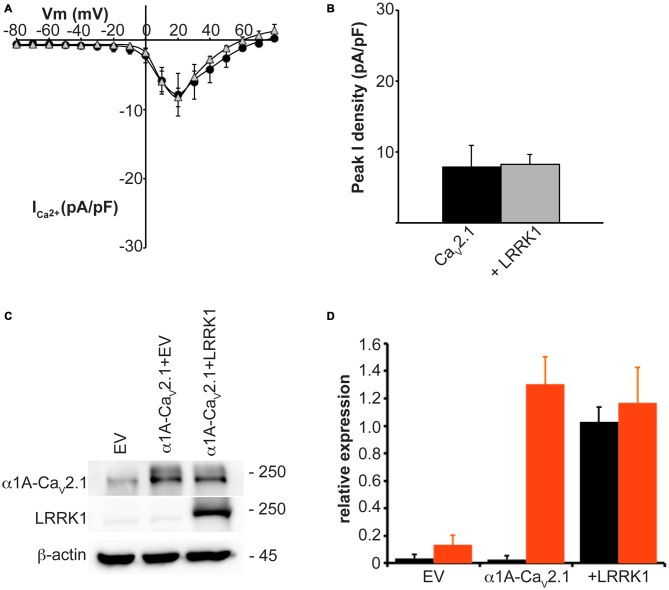
**LRRK1 has no effect on Ca_V_2.1 current density. (A)** Mean *I-V* relationships of peak Ca^2+^ current density from HEK293 cells transfected with Ca_V_2.1 alone (black circles; *n* = 10) or co-transfected with LRRK1 (gray triangles; *n* = 10 cells from four independent experiments). **(B)** Mean peak Ca^2+^ current density at a holding potential of 20 mV in cells expressing Ca_V_2.1 alone or co-expressing LRRK1. **(C)** Expression of myc-LRRK1, the Ca_V_2.1 α1A subunit and β-actin in HEK293 cells transfected with EV or Ca_V_2.1 channel subunits alone or plus myc-LRRK1 as evaluated by western-blotting. **(D)** Quantification of LRRK1 (black bars) and Ca_V_2.1 α1A subunit (red bars) protein level normalized to actin expression. Data are expressed as mean ± SEM, (*n* = 3).

In addition to LRRK2, humans and other mammals express a paralog protein, LRRK1, that has the closest sequence homology and shares similar domain structure to LRRK2. However, LRRK1 is not associated with Parkinson’s disease and has differential protein interactions suggesting it performs distinct cellular functions from LRRK2 (Reyniers et al., 2014). To evaluate whether LRRK2 effects on Ca_V_2.1 channel function were specific or could also be replicated by LRRK1, we measured whole cell Ca_V_2.1 properties in HEK293 cells co-transfected with LRRK1. Under these conditions, LRRK1 had no influence on current density across the entire voltage range that was tested (Figure 2A). Peak Ca_V_2.1 current density was not significantly different between cells transfected with all three Ca_V_2.1 channel subunits or co-transfected with LRRK1 (Figure 2B). To confirm exogenous LRRK1 and Ca_V_2.1 α1A subunit expression, the amount of LRRK1 and α1A protein relative to endogenous β-actin was measured via Western blot (Figure 2C). A robust expression of LRRK1 and α1A was detected in LRRK1 transfected cells compared to EV controls (Figure 2D). These results confirm that the effect of augmented Ca_V_2.1 current is specific to LRRK2. Despite being a homologous protein to LRRK2 in terms of sequence and structure, LRRK1 has no impact on Ca_V_2.1 channel function. It is therefore possible that the effects of LRRK2 on Ca_V_2.1 are mediated by unique LRRK2 domains and/or protein-protein interactions.

**Figure 3:**
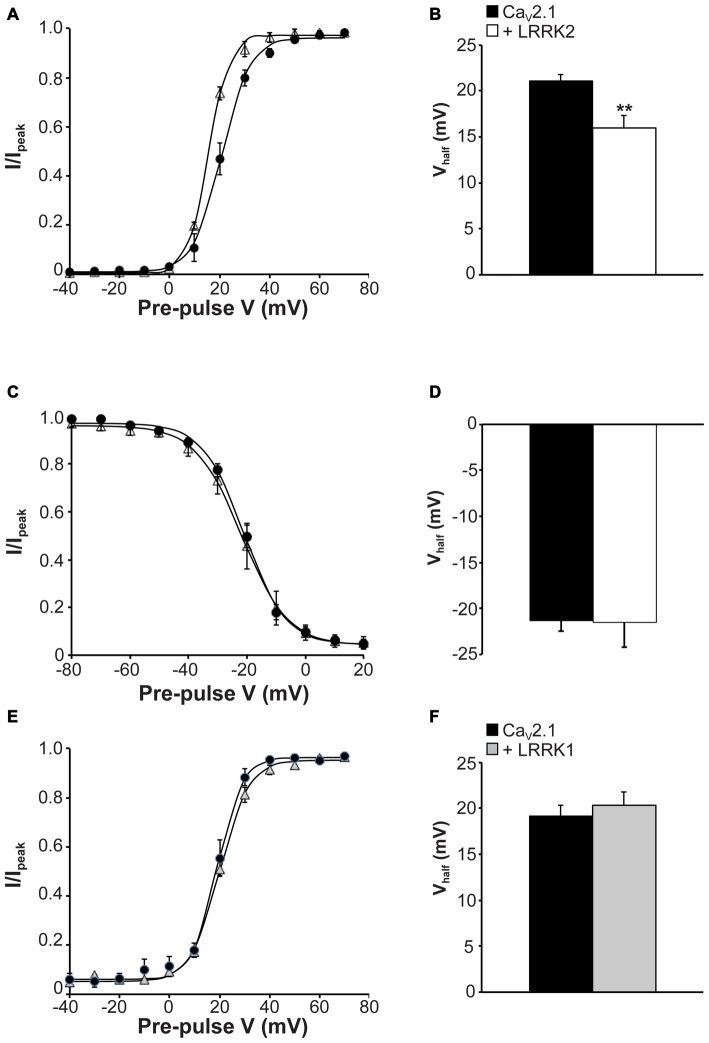
**Effect of LRRK2 on Ca_V_2.1 activation and inactivation properties. (A)** Voltage dependence of activation curves were recorded from HEK293 cells transfected with Ca_V_2.1 channels alone (black circles; *n* = 8 cells from four independent experiments) or co-transfected with LRRK2 (white triangles; *n* = 9 cells from four independent experiments). **(B)** LRRK2 significantly decreased the V_half_ of Ca_V_2.1 activation compared to control cells (where ** indicates *p* < 0.01; unpaired *t*-test).** (C)** Voltage dependence of steady-state inactivation curves from HEK293 cells transfected with Ca_V_2.1 channels alone (solid circles) or co-transfected with LRRK2 (open triangles; *n* = 6 cells from three independent experiments).** (D)** LRRK2 had no significant effect on the V_half_ of Ca_V_2.1 steady-state inactivation compared to control cells.** (E)** Voltage dependence of activation curves were recorded from HEK293 cells transfected with Ca_V_2.1 channels alone (black circles) or co-transfected with LRRK1 (gray triangles; *n* = 10 cells from four independent experiments). **(F)** LRRK1 had no significant effect on the V_half_ of Ca_V_2.1 activation compared to control cells.

The observed increase in Ca_V_2.1 current density with LRRK2 expression could be caused by LRRK2 increasing Ca_V_2.1 channel numbers at the membrane and/or direct effects of the kinase on Ca_V_2.1 channel gating. To provide evidence for the latter, we tested the effects of LRRK2 co-expression on the activation and inactivation gating properties of Ca_V_2.1. Figure 3A shows that LRRK2 caused a hyperpolarizing shift in the voltage-dependence of activation curve. This was associated with a significant hyperpolarization of the half-activation voltage (V_half_) from 21.08 ± 0.69 mV (*n* = 8) in control cells to 16.00 ± 1.33 mV (*n* = 9) in LRRK2 transfected cells (Figure 3B). This data supports a role for LRRK2 in directly regulating Ca_V_2.1 channel gating by increasing the proportion of activated channels at more hyperpolarized membrane potentials. In contrast, LRRK2 had no effect on inactivation of Ca_V_2.1. Voltage-dependent inactivation curves for Ca_V_2.1 were comparable between control and LRRK2 transfected cells (Figure 3C). Also, the half-inactivation voltage in control cells (−21.33 ± 1.13 mV, *n* = 6) was not significantly different from that of LRRK2 transfected cells (−21.54 ± 2.67 mV, *n* = 6; Figure 3D). In addition, we also investigated the effect of LRRK1 on activation properties. As LRRK1 did not significantly alter Ca_V_2.1 current density, we hypothesized that LRRK1 would not have any effect on Ca_V_2.1 voltage-dependent activation. Our results support this hypothesis showing that voltage-dependent activation curves do not differ between control and LRRK1 transfected cells (Figure 3E). Furthermore, no significant difference was detected in the half-activation voltage between control cells (−19.18 ± 1.18 mV, *n* = 8) and LRRK1 transfected cells (−20.36 ± 1.43 mV, *n* = 10; Figure 3F). Overall, these results indicate that LRRK2 regulates Ca_V_2.1 channel function via a specific effect on activation gating.

### Kinase Activity of LRRK2 is Required to Augment Ca_V_2.1 Function

**Figure 4:**
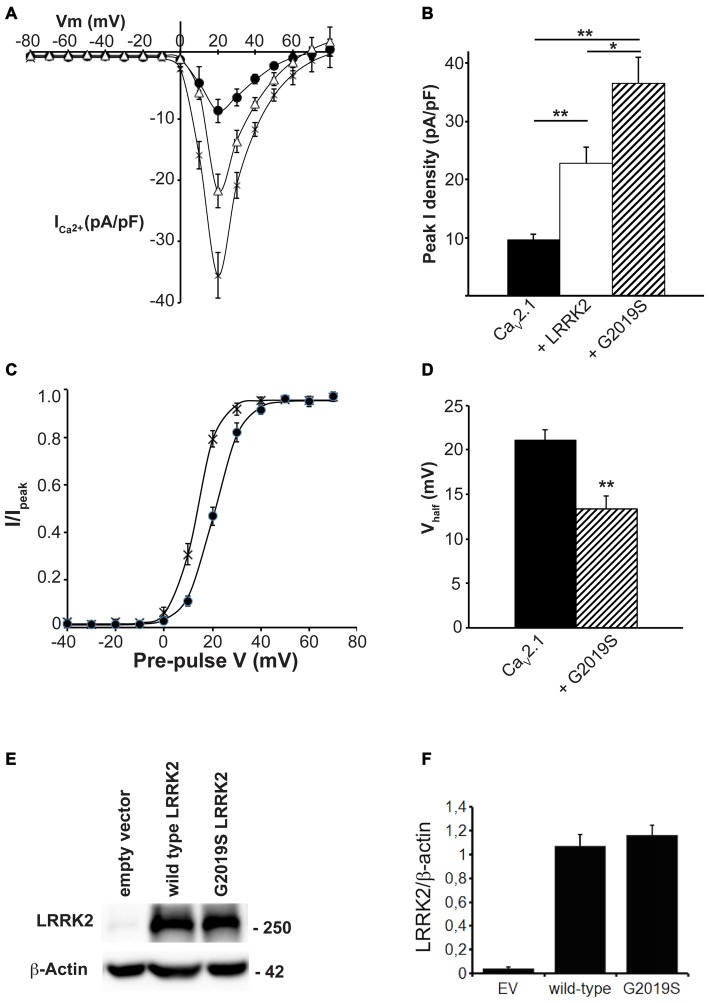
**The G2019S LRRK2 mutant causes an additional increase in Ca_V_2.1 current density. (A)** Mean *I-V* relationships of peak Ca^2+^ current density from HEK293 cells transfected with Ca_V_2.1 channels alone (solid circles; *n* = 10 cells from four independent experiments) or co-transfected with wt LRRK2 (white triangles; *n* = 9 cells from four independent experiments) or G2019S LRRK2 (crosses; *n* = 9 cells from four independent experiments). **(B)** Mean peak Ca^2+^ current density at a holding potential of 20 mV in cells expressing Ca_V_2.1 alone, co-expressing wt LRRK2 or co-expressing G2019S LRRK2.** (C)** Voltage dependence of activation curves were recorded from HEK293 cells transfected with Ca_V_2.1 channels alone (black circles) or co-transfected with G2019S LRRK2 (crosses; *n* = 9 cells from four independent experiments). **(D)** G2019S LRRK2 significantly decreased the V_half_ of Ca_V_2.1 activation compared to control cells (where ** indicates *p* < 0.01; unpaired *t*-test). **(E)** HEK293 were transfected with EV, Ca_V_2.1 channel subunits alone or plus wt LRRK2 or G2019S LRRK2. The expression of α1A, wt LRRK2 or G2019S LRRK2 was evaluated by western-blotting. **(F)** The graph reports LRRK2 (black bars) and Ca_V_2.1 α1A subunit (red bars) protein level normalized vs. actin level. Data are expressed as mean ± SEM, (*n* = 8).

Many of the physiological and pathophysiological roles of LRRK2 depend on its kinase activity. To determine whether the effects of LRRK2 on Ca_V_2.1 are linked to its kinase function, we used the LRRK2 kinase mutant G2019S that has increased kinase activity. This mutation enhances the ability of LRRK2 to phosphorylate both itself and other protein substrates (West et al., 2005; Martin et al., 2014). Therefore, if effects of LRRK2 on Ca_V_2.1 function require kinase activity, we hypothesized that the G2019S mutant would have enhanced effects on Ca_V_2.1 function. To test this hypothesis, we measured the Ca_V_2.1 current density in HEK293 cells co-transfected with wt or G2019S LRRK2. *I-V* relationships recorded from cells co-expressing the G2019S LRRK2 mutant exhibited an increase in Ca_V_2.1 current density in the voltage ranges of 10–50 mV compared to cells expressing only the three Ca_V_2.1 subunits (Figure 4A). Moreover, G2019S LRRK2 increases current density further than co-expression of wt LRRK2 (Figure 4A). Comparison of the peak Ca_V_2.1 current density in these three conditions confirms that G2019S LRRK2 significantly elevates Ca_V_2.1 current (−36.50 ± 4.45 pA/pF; *n* = 9) above both Ca_V_2.1 (−9.64 ± 0.90 pA/pF; *n* = 10) and wt LRRK2 (−22.72 ± 2.78 pA/pF; *n* = 9; Figure 4B). Since G2019S LRRK2 had a stimulatory effect on Ca_V_2.1 current density, we next examined whether this was also associated with changes to voltage-dependent activation properties. Figure 4C shows that the voltage-dependent activation curve for Ca_V_2.1 in the presence of G2019S has shifted to more hyperpolarized potentials compared to controls. G2019S caused a significant decrease in the half-activation voltage from control values of 20.11 ± 1.0 mV (*n* = 9) to 13.40 ± 0.61 mV (*n* = 9; Figure 4D). To verify that LRRK2 protein expression was similar between G2019S and wt and that it did not affect co-expression levels of Ca_V_2.1, we measured the levels of exogenous LRRK2 and α1A expression in HEK293 cells via Western blot. A representative blot is shown in Figure 4E which reveals that HEK293 cells transfected with EV have minimal LRRK2 protein. However, both wt and G2019S LRRK2 transfected cells have abundant and equivalent LRRK2 and α1A subunit expression. Normalized to β-actin levels, wt LRRK2 expression was not significantly different to G2019S expression (Figure 4F). Overall, these results indicate that increased kinase activity of LRRK2 enhances the stimulatory effect on Ca_V_2.1 channels, supporting a role for LRRK2 acting as a kinase to increase Ca_V_2.1 function.

**Figure 5:**
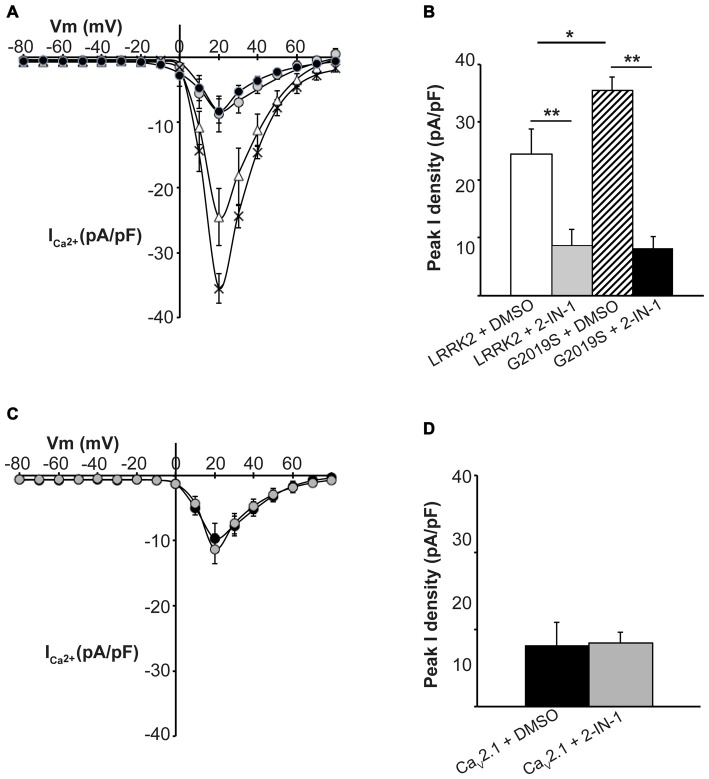
**Inhibition of LRRK2 prevents effect on Ca_V_2.1 current density. (A)** Mean *I-V* relationships of peak Ca^2+^ current density from HEK293 cells co-transfected with Ca_V_2.1 and LRRK2 pre-treated with vehicle (white triangles) or the LRRK2 inhibitor LRRK2-IN-1 (gray circles; *n* = 10 cells from four independent experiments). Also shown are mean *I-V* relationships of cells co-transfected with Ca_V_2.1 channels and G2019S pre-treated with vehicle (crosses) or LRRK2-IN-1 (black circles; *n* = 10 cells from four independent experiments). **(B)** LRRK2-IN-1 treated cells had a significantly reduced peak Ca^2+^ current density compared to vehicle (where ** indicates *p* < 0.01) while peak Ca^2+^ current density in G2019S cells was still significantly elevated over LRRK2 cells pre-treated with vehicle (where * indicates *p* < 0.05) and significantly reduced by LRRK2-IN-1 pre-treatment (where ** indicates *p* < 0.01; one-way ANOVA). **(C)** LRRK2-IN-1 pre-treatment (gray circles) had no effect on Ca_V_2.1 current density in the absence of exogenous LRRK2 expression compared to vehicle treated controls (black circles; *n* = 10 cells from four independent experiments). **(D)** Peak Ca^2+^ current density was comparable between LRRK2-IN-1 pre-treated cells compared to vehicle controls.

To further demonstrate that LRRK2 kinase activity is required to stimulate Ca_V_2.1, the effect of LRRK2 kinase inhibition was investigated. HEK293 cells co-expressing all three Ca_V_2.1 subunits with either wt or G2019S LRRK2 were pre-treated with LRRK2-IN-1, an inhibitor of both wt and G2019S LRRK2 kinase activity (Deng et al., 2011). Whole cell Ca_V_2.1 current density was then measured and compared to control cells pre-treated with DMSO vehicle. LRRK2 transfected cells pre-treated with vehicle had similar increases in Ca_V_2.1 current density (Figure 5A) to LRRK2 alone (Figure 1C) demonstrating DMSO did not impact on the stimulatory effect of LRRK2. In contrast, cells that had been pre-treated with LRRK2-IN-1 had reduced Ca_V_2.1 current densities (Figure 5A) which were comparable to cells expressing Ca_V_2.1 alone (Figure 1C). LRRK2-IN-1 pre-treatment caused a significantly reduced peak Ca_V_2.1 current density of −8.74 ± 2.71 pA/pF (*n* = 10) compared to −24.53 ± 4.36 pA/pF (*n* = 10) in vehicle pre-treated controls (Figure 5B).

We also investigated whether LRRK2-IN-1 could reverse the stimulatory effects of the G2019S mutant on Ca_V_2.1 function. Figure 5A demonstrates that whole cell Ca_V_2.1 current density in G2019S LRRK2 transfected cells pre-treated with LRRK2-IN-1 was reduced between the voltage range of 10–60 mV compared to vehicle treated cells. A significant difference in peak Ca_V_2.1 current density was observed between LRRK2-IN-1 (−8.29 ± 1.86 pA/pF, *n* = 10) and vehicle (−35.51 ± 2.25 pA/pF, *n* = 10) pre-treated cells (Figure 5B). Taken together, these results indicate that the stimulatory effects of LRRK2 on Ca_V_2.1 channels are dependent on the ability of LRRK2 to act as a kinase.

Importantly, LRRK2-IN-1 had no effect on Ca_V_2.1 current density in the absence of LRRK2 co-transfection as mean Ca_V_2.1 current density was similar between cells tranfected with Ca_V_2.1 channels and pre-treated with LRRK2-IN-1 or DMSO (Figures 5C,D). This confirms that the effects of the inhibitor are specific to LRRK2 and are not caused by non-specific effects on the channel directly or other channel regulatory proteins.

**Figure 6:**
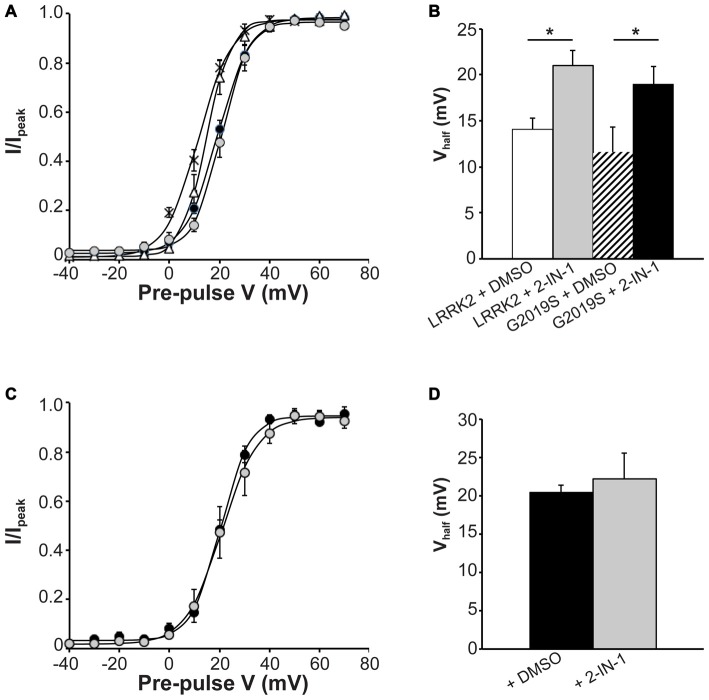
**Effect of LRRK2-IN-1 on Ca_V_2.1 activation properties. (A)** Voltage dependence of activation curves were recorded from HEK293 cells transfected with Ca_V_2.1 channels plus LRRK2 and pre-treated with LRRK2-IN-1 (gray circles) or DMSO vehicle (white triangles; *n* = 9 cells from four independent experiments). A separate group was co-transfected with G2019S and pre-treated with LRRK2-IN-1 (black circles) or DMSO (crosses; *n* = 9 cells from four independent experiments). **(B)** LRRK2-IN-1 significantly increased the V_half_ of Ca_V_2.1 activation in both LRRK2 and G2019S cells compared to control cells (where * indicates *p* < 0.05; one-way ANOVA).** (C)** Voltage dependence of activation curves from HEK293 cells transfected with Ca_V_2.1 channels alone and pre-treated with DMSO (solid circles) LRRK2-IN-1 (gray circles; *n* = 10 cells from four independent experiments).** (D)** LRRK2-IN-1 had no significant effect on the V_half_ of Ca_V_2.1 activation compared to control cells.

Next, we investigated the effect that LRRK2-IN-1 had on activation kinetics of Ca_V_2.1 channels in cells transfected with LRRK2 and G2019S. Figure 6A illustrates that LRRK2-IN-1 shifts the voltage-dependent activation curves of both LRRK2 and G2019S cells to more depolarized potentials compared to DMSO treated controls. In cells transfected with LRRK2 and pre-treated with DMSO, the mean half-activation voltage was 14.08 ± 1.2 mV (*n* = 9), significantly more hyperpolarized than the value of 20.98 ± 1.7 mV (*n* = 9) obtained from LRRK2-IN-1 pre-treated cells (Figure 6B). In addition, LRRK2-IN-1 pre-treatment significantly depolarized the half-activation voltage of G2019S transfected cells to 22.24 ± 3.3 mV (*n* = 8) from a mean value of 11.60 ± 2.7 mV (*n* = 9; Figure 6B). This data indicates that phosphorylation activity of LRRK2 is required to affect changes in activation gating of the Ca_V_2.1 channel. To rule out the possibility that LRRK2-IN-1 acts directly on the Ca_V_2.1 channel to alter gating, we examined the effect of LRRK2-IN-1 on voltage-dependent activation of Ca_V_2.1 channels in the absence of exogenous LRRK2 expression. Results from these experiments show that LRRK2-IN-1 has no effect on either the voltage-dependent activation curve (Figure 6C) or the half-inactivation voltage (Figure 6D). Overall, these results indicate that the kinase activity of LRRK2 is required to mediate changes in the activation gating of Ca_V_2.1 channels.

### LRRK2 Interacts with Ca_V_2.1 Channels

**Figure 7:**
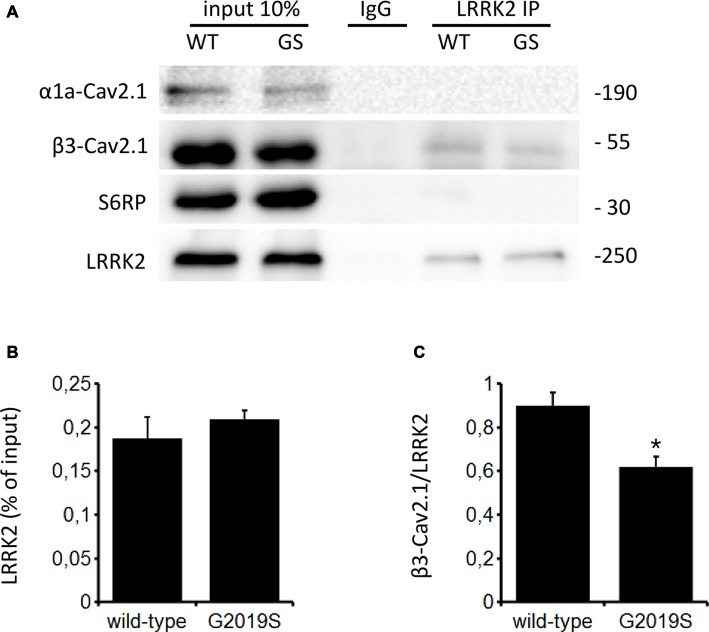
**LRRK2 interacts with the Ca_V_2.1 channel. (A)** Mouse forebrain specimens obtained from littermate wt and heterozygous BAC hG2019S (GS) mice were cross-linked and then processed for immunoprecipitation with rat anti LRRK2 antibodies. Eluted proteins were resolved by SDS-PAGE and detected with anti LRRK2, anti Ca_V_2.1 β3 subunit antibodies, anti Ca_V_2.1 α1a and anti S6 ribosomal protein (S6RP).** (B,C)** The graphs report the yield of protein recovered upon LRRK2 immunoprecipitation expressed as a percentage of relative input **(B)** and as amount of pulled Ca_V_2.1 β3 subunit relative to the amount of pulled LRRK2 **(C)**. Bars represent mean ± SEM (*n* = 4) where * indicates *p* < 0.05; unpaired *t*-test.

The function of Ca_V_ channels can be modulated by regulatory proteins directly interacting with channel subunits (Kiyonaka et al., 2007; Jiang et al., 2008). Since LRRK2 has been shown to interact with a number of presynaptic proteins (Piccoli et al., 2011, 2014), we investigated whether LRRK2 interacts with Ca_V_2.1 channel subunits as a mechanism to control channel function. To this aim, we immunoprecipitated endogenous LRRK2 from protein extracted from wt mouse forebrain specimen previously exposed to a cross-linking protocol. Interestingly, we noticed that rat anti LRRK2 but not generic rat IgG precipitates endogenous LRRK2 and the Ca_V_ channel β3 subunit but not the Ca_V_ channel α1 subunit or SP6 ribosomal protein (Figure 7A). We obtained a similar outcome by immunoprecipitating LRRK2 protein from mouse forebrain specimen obtained from BAC hG2019S LRRK2 mice indicating that β3 interacts also with G2019S LRRK2, even though to a significantly reduced extent compared to wt LRRK2 (Figures 7B,C). All together, these data suggest that LRRK2 specifically interacts with the Ca_V_2.1 channel β3 subunit.

### Endogenous Ca_V_ Channel Function is Also Increased by LRRK2

**Figure 8:**
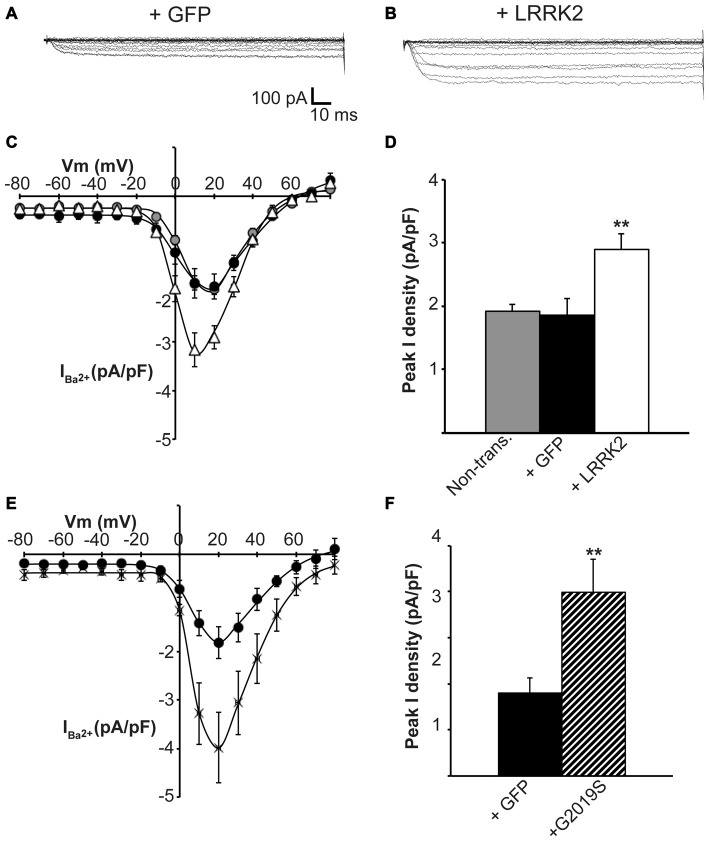
**Endogenous Ca_V_ channels are stimulated by LRRK2 in PC12 cells.** Representative whole cell Ba^2+^ currents recorded from PC12 cells transfected with GFP alone **(A)** or together with LRRK2 **(B). (C)** Mean *I-V* relationships of peak Ba^2+^ current density from non-transfected (gray circles), GFP transfected (black circles) or LRRK2 transfected PC12 cells (white triangles; *n* = 7–9 from four independent experiments). **(D)** Mean peak Ba^2+^ current density at a holding potential of 20 mV in PC12 cells transfected as described in **(C)** where ** indicates *p* < 0.01, one-way ANOVA. **(E)** The effect of G2019S LRRK2 (crosses) on Ca_V_ current density compared to GFP controls (circles; *n* = 10 cells from four independent experiments).** (F)** Peak Ba^2+^ current density was significantly elevated in G2019S LRRK2 cells compared to vehicle controls (where ** indicates *p* < 0.01; unpaired *t*-test).

**Figure 9:**
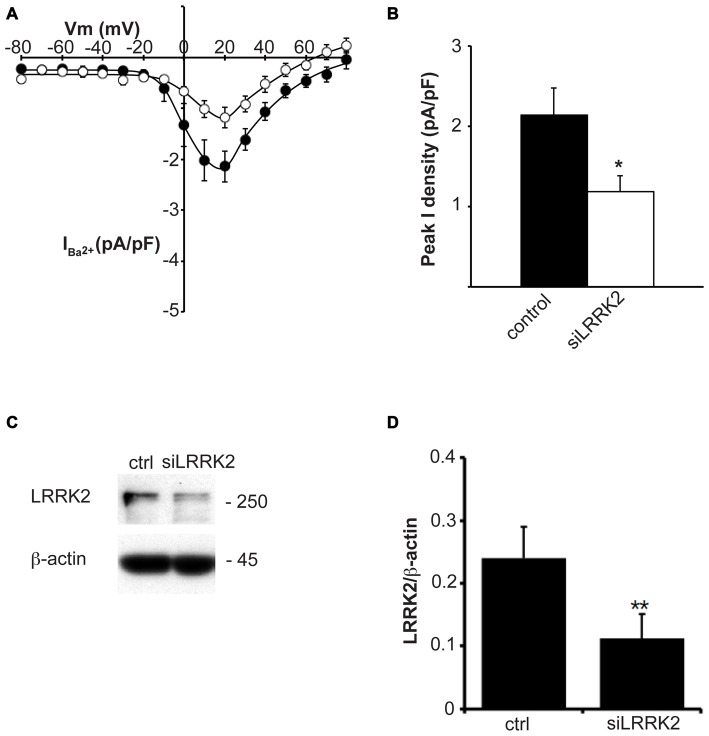
**Knockdown of endogenous LRRK2 in PC12 cells decreases native CaV channel function. (A)** Silencing LRRK2 (white circles) reduces Ca_V_ current density compared to LVTH controls (solid circles; *n* = 10 cells from four independent experiments).** (B)** Peak Ba^2+^ current density was significantly decreased in siLRRK2 cells compared to controls (where * indicates *p* < 0.05; unpaired *t*-test). **(C)** PC12 cells were transfected with LVTH control (ctrl) vector or siLRRK2 vector and the expression of LRRK2 and β-actin was evaluated by western blotting. **(D)** The graph reports the LRRK2 protein level normalized to β-actin. Data are expressed as mean ± SEM (*n* = 4; where ** indicates *p* < 0.01; unpaired *t*-test).

Having established that LRRK2 regulates the function of recombinant Ca_V_2.1 channels, we next investigated whether native Ca_V_ channels were also subjected to regulation by LRRK2 using the neuroendocrine rat pheochromocytoma PC12 cell line. PC12 cells endogenously express a diversity of Ca_V_ channels including Ca_V_1.2, Ca_V_1.3, Ca_V_2.1 and Ca_V_2.2 (Plummer et al., 1989; Liu et al., 1996). To determine whether these native Ca_V_ channels were modulated by LRRK2, we transfected PC12 cells with wt LRRK2 plus GFP or GFP alone. Ba^2+^ was used as the charge carrier to measure Ca_V_ currents in PC12 cells given its higher permeability since endogenous levels of Ca_V_ channels were expected to be much smaller than in our heterologous expression system. Indeed, representative Ba^2+^ current traces recorded from PC12 cells show that GFP-transfected cells have small inward Ba^2+^ currents (Figure 8A). However, these currents still exhibit the characteristic steep increase between the voltages of −10 to 40 mV typical of high voltage activated Ca^2+^ channels (Figure 8B). LRRK2 transfection increased the relative amount of current (Figures 8B–D) in this voltage range while GFP transfection had no effect compared to non-transfected controls (Figure 8D). Interestingly, LRRK2 transfection also caused a hyperpolarizing shift in the voltage at which the peak inward Ba^2+^ current was recorded, from −20 to −10 mV (Figure 8B). On average, LRRK2 transfection caused a significant increase in peak current density at 20 mV from −1.86 ± 0.26 pA/pF (*n* = 9) in GFP transfected cells to −2.90 ± 0.24 pA/pF (*n* = 7) in LRRK2 cells (Figure 8C). No significant difference in peak Ba^2+^ current density was detected between non-transfected and GFP transfected cells (Figure 8C). To determine whether G2019S LRRK2 also stimulates endogenous Ca_V_ channels, whole cell Ba^2+^ currents were measured in PC12 cells transfected with GFP or G2019S plus GFP. Similar to the effect of wt LRRK2, G2019S caused a large increase in current compared to controls (Figure 8E). In contrast however, there was no apparent shift in the voltage at which peak Ba^2+^ current was recorded (Figure 8E). This peak current density was at 20 mV where G2019S transfection caused an increase in current from −1.81 ± 0.33 pA/pF (*n* = 10) in GFP transfected cells to −3.98 ± 0.73 pA/pF (*n* = 10; Figure 8F). Finally, to investigate a role for endogenous LRRK2 in regulating native Ca_V_ channels in PC12 cells, we transfected cells with a LRRK2 silencing construct we had previously used to knockdown LRRK2 in cortical neurons (Piccoli et al., 2011). Figure 9A demonstrates that siLRRK2 cells had reduced Ba^2+^ currents across several voltages compared to control cells transfected with the EV as performed previously. Peak Ba^2+^ currents at 20 mV were significantly reduced in siLRRK2 cells (Figure 9B) indicating that endogenous LRRK2 has a physiological role in controlling activity of native Ca_V_ channels in PC12 cells. Endogenous LRRK2 protein expression was confirmed in control PC12 cells (Figure 9C) which was significantly reduced in siRNA treated cells (Figure 9D). Taken together, these results suggest that, in addition to stimulating recombinant Ca_V_2.1 channels, LRRK2 is able to up-regulate activity of native Ca_V_ channels. The fact that PC12 cells express a variety of different Ca_V_ channel classes suggests that the effect of LRRK2 is not limited to Ca_V_2.1 alone but may also stimulate other Ca_V_ channel types.

## Discussion

Ca_V_2.1 channel activity is regulated by a diversity of kinases and other signaling proteins to control Ca^2+^ influx and subsequent Ca^2+^-dependent cellular functions. Here, we demonstrate for the first time that LRRK2 can modulate Ca_V_2.1 channel function by causing a large increase in Ca^2+^ current. This effect was associated with altered Ca_V_2.1 activation properties and was dependent on the kinase activity of LRRK2. These functional effects may be a consequence of a physical interaction between LRRK2 and the β3 subunit. Furthermore, LRRK2 also up-regulated endogenous Ca_V_ channel activity suggesting that LRRK2 may have broad effects on a variety of different Ca_V_ channels. Altogether, these results describe a novel regulatory mechanism of Ca_V_2.1 channels that is likely to play important roles in both the physiology and pathophysiology of cellular Ca^2+^ signaling.

Several protein kinases have been shown to target Ca_V_2.1 channels to modulate Ca^2+^ entry. PKC phosphorylates the I-II linker region of the Ca_V_2.1 α1A subunit to increase Ca_V_2.1 current by antagonizing G-protein inhibition of the channel (Zamponi et al., 1997). CaMKII also enhances Ca^2+^ entry but does so by directly binding to the C-terminus of Ca_V_2.1 to slow inactivation kinetics and cause a depolarizing shift in the voltage-dependence of inactivation (Jiang et al., 2008). In contrast, both Cdk-5 (Tomizawa et al., 2002) and GSK-3 (Zhu et al., 2010) have a negative effect on Ca_V_2.1 function by phosphorylating the II-III loop of Ca_V_2.1 α1A subunit to prevent SNARE protein interactions. Therefore, kinases utilize a range of mechanisms to exert either stimulatory or inhibitory effects on Ca_V_2.1 function. In the present study, we have demonstrated that LRRK2 stimulates Ca_V_2.1 channels causing an increase in Ca^2+^ current density. The mechanism behind this effect appears to be due to a direct effect of LRRK2 on modulating channel gating. This direct action of LRRK2 is supported by our results showing that voltage-dependent activation is significantly shifted to more hyperpolarized potentials with LRRK2 overexpression. This would increase the proportion of active channels at more hyperpolarized voltages, leading to an increased current density. This indicates that LRRK2 is impacting how the channel changes conformation at the membrane in response to voltage; an effect most likely to be caused by LRRK2 interacting with the macromolecular Ca_V_2.1 complex. Moreover, our co-immunoprecipitation results reveal that LRRK2 interacts with the Ca_V_2.1 β3 subunit. Although it is possible this could involve indirect associations via secondary or tertiary binding relationships, this is unlikely here as we were unable to co-immunoprecipitate the β3 interacting α1A subunit. Therefore, since β3 subunits significantly affect voltage-dependent activation (Sandoz et al., 2001; He et al., 2007), we propose that LRRK2 is exerting an effect on this mode of Ca_V_2.1 channel gating through its interaction with the β3 subunit. This is in agreement with the effects of another presynaptic protein, RIM1 (Kiyonaka et al., 2007), which has been shown to interact with Ca_V_ β subunits to augment Ca_V_ channel function. Interestingly, the effects of RIM1 binding on Ca_V_2.1 properties depended on the type of β subunit expressed. With β3, RIM1 slowed inactivation kinetics and shifted the voltage-dependence of inactivation but had no effect on current density or voltage dependence of activation (Kiyonaka et al., 2007). This is in agreement with our findings of a β interacting protein altering some channel functions but not others. Taken together, our results support a direct role of LRRK2 as a Ca_V_ channel regulatory protein interacting with the β3 subunit to alter gating as a means to enhance Ca_V_2.1 function.

Alternatively, LRRK2 could exert an increase in Ca_V_2.1 current density by increasing channel numbers at the membrane. Increasing the translation and synthesis of Ca_V_2.1 channel subunits is one potential mode for how LRRK2 might influence channel numbers. This is supported by a recent study showing LRRK2 phosphorylates the ribosomal protein s15 to increase mRNA translation (Martin et al., 2014). In addition, increased Ca_V_2.1 channel numbers may be caused by LRRK2 regulating trafficking pathways responsible for determining the amount of Ca_V_2.1 at the plasma membrane. In this scenario, LRRK2 interactions with the β subunit could be important as trafficking of the Ca_V_ channel complex from the endoplasmic reticulum to the plasma membrane is dependent on β subunits (Altier et al., 2011; Waithe et al., 2011). Furthermore, LRRK2 has been shown to affect a variety of intracellular trafficking pathways to control the membrane expression of several different cell surface proteins (Migheli et al., 2013; Cho et al., 2014; Gómez-Suaga et al., 2014). However, given that G2019S LRRK2 seems to bind the Ca_V_2.1 β3 subunit to a lesser extent, channel trafficking may not be the main target of LRRK2 activity.

A further insight into a potential mechanism is provided by our results which demonstrate that LRRK2 is required to phosphorylate a substrate in order to regulate Ca_V_2.1 activity. The G2019S LRRK2 mutant that has enhanced kinase activity caused a more pronounced effect on Ca_V_2.1 function while blocking both wt and mutant LRRK2 kinase activity with a LRRK2 inhibitor reversed these stimulatory effects. One possible interpretation of these results is that LRRK2 needs to autophosphorylate to become activated and initiate downstream effects on Ca_V_2.1 channels. This is supported by recent studies demonstrating that LRRK2 autophosphorylation is required for activation of LRRK2 GTPase activity that then influences interactions with LRRK2 substrates (Cirnaru et al., 2014; Law et al., 2014; Liu et al., 2016). Alternatively, LRRK2 could be directly phosphorylating Ca_V_2.1 proteins to alter channel function. Indeed, two of the Ca_V_2.1 channel subunits used in this study (α1A and β3) have been shown to be directly phosphorylated by other serine/threonine kinases (Zamponi et al., 1997; Grueter et al., 2008; Jiang et al., 2008). A direct role for LRRK2 phosphorylating Ca_V_2.1 subunits is more likely than indirect effects on Ca_V_ regulatory proteins given that experiments were performed in a heterologous expression system, however, further investigation is required to precisely identify substrates of LRRK2 phosphorylation that lead to increased Ca_V_2.1 channel function.

While both wt LRRK2 and the G2019S mutant appear to up-regulate Ca_V_2.1 function by the same mechanism, this was a specific effect of this particular kinase as we found that LRRK1 had no effect on Ca_V_2.1 current. Therefore, the fact that LRRK1 and LRRK2 share similar leucine-rich-repeat, GTPase and kinase domains but have divergent effects on Ca_V_2.1 channels indicates that the mode of regulation must be uniquely intrinsic to LRRK2 and is not a general effect of a common domain interaction. This is in agreement with other LRRK2 specific effects where increasing evidence suggests these two related proteins perform distinct cellular functions by interacting with different proteins (Reyniers et al., 2014).

Interestingly, the effect of LRRK2 may not be specific to Ca_V_2.1 channels only. We found that LRRK2 was able to up-regulate endogenous Ca_V_ current in PC12 cells that contain a variety of different types of Ca_V_ channels. Within this background of multiple types of Ca_V_ channel subunits, it was not possible here to discern if LRRK2 mediated increases in PC12 Ca_V_ current density involved alterations to activation gating. However, these results provide evidence that several types of Ca_V_ channels could be targeted by LRRK2. Future studies are required to identify which other types of Ca_V_ channels are targeted by LRRK2, the subunits involved and whether LRRK2 utilizes a common mechanism to upregulate Ca_V_ function as we have described here. If so, this would suggest that LRRK2 is involved in a general physiological role of stimulating Ca_V_ channel function to influence intracellular Ca^2+^ signaling. This could have important downstream effects on many Ca^2+^ dependent cellular processes such as vesicle trafficking and autophagy. In fact, accumulating evidence describes a role for LRRK2 at the presynaptic site. For example, we have previously shown that LRRK2 influences neurotransmission acting as a scaffolding element (Piccoli et al., 2011, 2014) as well as a kinase (Cirnaru et al., 2014). If, as we have shown here in a recombinant system, synaptic calcium channels are upregulated by LRRK2, it might be expected that stimulus evoked neurotransmitter release would be increased by LRRK2 activity and further by gain-of-function mutations such as G2019S. Notably, complementary G2019S models suggest that the gain-of-function LRRK2 mutation positively modulates presynaptic release; in particular, LRRK2 knock-in mouse G2019S mutants are characterized by elevated glutamate release and synaptic transmission (Beccano-Kelly et al., 2014), primary cultures from BAC hG2019S mice demonstrate increased synaptic vesicle trafficking (Belluzzi et al., 2016) and G2019S over-expression in PC12 cells increases dopamine release (Migheli et al., 2013). Indeed, we and others have shown that reducing LRRK2 expression has a similar impact on presynaptic properties (Piccoli et al., 2011; Matta et al., 2012) with contrasting effects also reported (Arranz et al., 2015). However, it is possible that LRRK2 plays different roles while acting as a scaffold or a kinase. An intriguing hypothesis is that LRRK2 may organize a molecular hub that impairs neurotransmission by tethering synaptic vesicles via protein-protein interactions (Piccoli et al., 2014). LRRK2 kinase activity may instead promote synaptic vesicle fusion via phosphorylation of presynaptic substrates such as snapin (Yun et al., 2013), EndophilinA (Matta et al., 2012) N-ethylmaleimide sensitive fusion (NSF; Belluzzi et al., 2016) and Ca_V_2.1 channels (this study). Given this dual role, any perturbation to LRRK2 levels or kinase activity may influence LRRK2 control of synaptic calcium channels and vesicle dynamics and, ultimately, on downstream neurotransmission.

The effects of LRRK2 on Ca_V_ channels may be part of a broader role of LRRK2 in regulating Ca^2+^ signaling proteins as LRRK2 has been shown to activate NAADP receptors to cause Ca^2+^ efflux from Ca^2+^ stores (Gómez-Suaga et al., 2012) and stimulate Na^+^/Ca^2+^ exchanger activity (Yan et al., 2015). In addition to a potential role in normal Ca^2+^ signaling, our results may also provide further insight into LRRK2 pathophysiology. The G2019S LRRK2 mutation associated with Parkinson’s disease stimulated Ca_V_2.1 channels to a greater degree than wt. This mutation has also been shown to cause mitochondrial degradation in cortical neurons which could be prevented by blocking Ca^2+^ influx via Ca_V_ channels (Cherra et al., 2013). Therefore, LRRK2 mutations could disrupt normal Ca^2+^ signaling as an early cellular event in the development of Parkinson’s disease.

In conclusion, this study has identified a novel role for LRRK2 in regulating Ca_V_2.1 channel function. Both wt and mutant LRRK2 augment Ca_V_2.1 function that is dependent on kinase activation. This was associated with LRRK2 interacting with the Ca_V_ β3 subunit. Although further investigation is required to determine whether this involves LRRK2 directly phosphorylating Ca_V_ subunits or having an indirect effect on channel regulatory proteins, our results provide further evidence that LRRK2 has an important regulatory role in cellular Ca^2+^ signaling.

## Author Contributions

CB, CS, MP-C and SBC performed experiments; CB, MP-C, GP and SBC analyzed data; GP and SBC designed experiments and wrote the article. All authors revised the manuscript and approved the final version.

## Conflict of Interest Statement

The authors declare that the research was conducted in the absence of any commercial or financial relationships that could be construed as a potential conflict of interest.
